# Long-term outcomes of intravitreal anti-VEGF therapies in patients affected by neovascular age-related macular degeneration: a real-life study

**DOI:** 10.1186/s12886-021-02055-6

**Published:** 2021-08-14

**Authors:** Paolo Corazza, Francesco Maria D’Alterio, Jamil Kabbani, Mostafa Mohamed Ragheb Alam, Stefano Mercuri, Harry Otway Orlans, Saad Younis

**Affiliations:** 1 Western Eye Hospital, Imperial College Healthcare NHS Trust, 171 Marylebone Rd, London, NW1 5QH UK; 2 Imperial College Ophthalmology Research Group (ICORG), Imperial College, London, UK; 3 Imperial College, London, UK; 4 Tanta University, Tanta, Egypt

**Keywords:** Anti-VEGF therapy, Choroidal neovascularization, Treat and extend, Wet-AMD, Real life data, Intravitreal injections

## Abstract

**Purpose:**

To describe real-life data from wet age-related macular degeneration (AMD) patients treated with anti-vascular endothelial growth factors (VEGFs) and to compare our results with previous studies and clinical trials.

**Methods:**

This retrospective monocentric cohort study analyzed 865 eyes of 780 wet-AMD patients treated with an anti-VEGF treat-and-extend regimen over a long-term follow-up period. Aflibercept and Ranibizumab were considered first-line agents whereas Bevacizumab was reserved for use on a compassionate basis in patients not meeting treatment criteria. All patients underwent a best corrected visual acuity (BCVA) assessment at each follow-up visit.

**Results:**

One-year follow-up figures were available for 82.5% of patients, whilst follow-up data was recorded for 55.6%, 37.6%, 25.1%, and 15.0% of the cohort at years 2, 3, 4, and 5 respectively. Patients treated with Bevacizumab received fewer yearly injections than those treated with Ranibizumab. However, no significant difference in the number of injections per year was detected in other comparisons between groups. Whilst our data showed no significant difference in mean BCVA between the three groups, there was a gradual deterioration of visual function over time for the patient cohort as a whole.

**Conclusion:**

No significant differences between the 3 anti-VEGF molecules were recorded in wet-AMD patients in real-life conditions. Despite the long-term therapy, we found a slight reduction in visual function especially after the third year of treatment.

## Introduction

Age-related macular degeneration (AMD) is currently considered to be one of the chief causes of irreversible visual loss in developed countries, with an estimated 7% of global blindness being attributable to this condition [1–3]. The prevalence of AMD is increasing due improvements in life expectancy and earlier detection of the disease. Late stages of the disease are typically characterized by the loss of central vision due to atrophic changes and/or the presence of neovascularization arising from the choroidal, or occasionally from the retinal, vascular network [4]. The wall structure of these new vessels is abnormal, and their presence is frequently associated with leakage and/or exudation within or beneath the retina. The importance of vascular endothelial growth factor (VEGF) in the pathogenesis of neovascular AMD was first established in 1983 [5], and since then, numerous studies have confirmed the central role of this protein in the disease process [6, 7]. Three VEGF-targeting molecules have gained worldwide acceptance for intravitreal use in the treatment of neovascular AMD: Bevacizumab, a recombinant humanized monoclonal antibody; Ranibizumab, a monoclonal antibody fragment; and Aflibercept, a recombinant fusion protein consisting of the binding domains of VEGF receptors 1 and 2 together with the Fc portion of the human IgG1 immunoglobulin. Together these molecules represent the first-line therapy for wet AMD [8], with many large-scale and well-run clinical trials demonstrating their safety and efficacy [9–11]. Recently, Brolucizumab has been developed as a new long-acting anti-VEGF agent and clinical trials are underway to define its efficacy and safety profile [12]. Clinical trial results may not, however, be replicable in real-world practice where patients frequently fall outside of the rigid inclusion or exclusion criteria used in the design of such trials and are exposed to a less intensive course of treatment. The importance of real-world data in wet AMD treatment is now widely recognized and this has become an intense field of research in recent years. Both the European Medicines Agency (EMA) and the US Food and Drug Administration (FDA) have defined such data as that coming from aggregation and analysis of data from clinical experience, outside of randomized clinical trials [13, 14]. The purpose of our study is to describe real-world outcomes in wet AMD patients treated with anti-VEGF therapy over a long follow-up period, and to compare these results with those published by other centers and with clinical trial data.

## Methods

This real-life retrospective monocentric cohort study was conducted at a tertiary referral centre, the Western Eye Hospital, part of the Imperial College Healthcare NHS Trust in London, United Kingdom. This study was performed in accordance with the Declaration of Helsinki and was approved by the local Institutional Review Board. 865 eyes of 780 patients were analyzed. All patients were treated for wet AMD in our one-stop Macular Clinic with a treat-and-extend (TEX) regimen in accordance with the National Institute for Health and Care Excellence (NICE), UK guidelines (Table 1) [15, 16]. We considered Afilbercept and Ranibizumab as first-line agents for newly diagnosed wet AMD patients. The decision to use Ranibizumab or Aflibercept was randomized and made regardless of the patient's clinical and OCT findings. In bilateral cases, the same anti-VEGF agent was used for both eyes. Our TEX protocol for Ranibizumab and Aflibercept is summarized as follows: new patients began the treatment with a loading phase of three monthly injections, the treatment was then continued on a monthly basis for Ranibizumab (every 4 weeks) and on a bimonthly basis for Aflibercept (every 8 weeks) until any macular hemorrhages previously seen on slit lamp biomicroscopic examination had disappeared, and intraretinal fluid (IRF) and/or subretinal fluid (SRF) on optical coherence tomography (OCT) had resolved or was stable compared to the previous two visits. The treatment interval was then sequentially lengthened by 2 weeks at each visit if there were no signs of disease activity, up to 12 weeks. The follow-up period was shortened to 4 weeks if any sign of exudation or new macular hemorrhage was evident clinically or on OCT. The treatment interval was also shortened if the patient had a subjective decline in vision of 5 letters or more as measured using the Early Treatment Diabetic Retinopathy Study (ETDRS) chart. The treatment was discontinued and the patient discharged if no signs of activity noticed for 3 consecutive visits at 12 weeks interval and/or if any permanent structural damage noticed (e.g. macular atrophy, disciform scar, macular fibrosis). The use of Bevacizumab was usually considered on a compassionate basis in patients with single functioning eyes who did not meet the NICE treatment criteria for use of Ranibizumab or Aflibercept (i.e. visual acuity recorded as less than 6/96 or more than 6/12). Data was collected retrospectively through Electronical Medical Records software (EMR) (Medisoft, Medisoft Limited, Leeds, UK) in a similar manner to that described in other research [17–19]. Patients recorded with a new diagnosis of wet AMD who attended the macular clinic between December 2006 and April 2019 were included. Due to the real-life nature of the study, we included patients until all data needed was available, or loss of follow up and/or a treatment stop was recoded. Other inclusion criteria were: diagnosis of wet AMD with choroidal neovascularization (CNV) confirmed with optical coherence tomography angiography (OCTA) or fluorescein angiography (FA), best correct visual acuity (BCVA) ≤ 6/12 and ≥ 6/96, no previous ocular treatment, and absence of other systemic or ocular pathologies that could compromise visual function, such as ischemic optic neuropathy or glaucoma. In addition, pregnant women and patients who suffered from a stroke or heart attack in the previous 6 months were excluded from treatment, as were eyes that switched treatment molecules during the observational period.

**Table 1:** A summary of NICE (National Institute for Health and Care Excellence) guidelines for anti-VEGF treatment in Wet AMD

Indication	• Wet AMD
Clinical and imaging features	• Best-corrected visual acuity between 6/12 and 6/96 • Evidence of disease activity • Absence of permanent structural damage to the central fovea • Lesion size ≤ 12 disc areas

The baseline was considered as the time of diagnosis of wet AMD, and anti-VEGF treatment was started on the same day. At every visit all patients underwent a BCVA recording using ETDRS chart, intraocular pressure (IOP) measurement with an iCARE tonometer, OCT of the macular region (with macular dense and 7 line scans) using the Spectralis HRA + OCT platform (Heidelberg Engineering, Heidelberg, Germany), and clinical examination of the posterior pole with a 66D or 90D indirect fundus-viewing lens. We considered patients with stable visual acuity as those who did not lose more than 5 ETDRS letters from baseline at each year's time-point thereafter. Our definition of reduced VA was a loss of 15 or more ETDRS letters (0.3 LogMAR) from baseline, and for improvement in VA, a gain of 15 letters or more (0.3 LogMAR).

### Statistical analysis

All statistical analyses were conducted using Prism software (Version 8.1.1, GraphPad Software, Inc., San Diego, CA), with BCVA recorded in LogMAR for analysis purposes. Comparisons between mean BCVA for patient groups treated with different drugs were made using two-way ANOVA testing with drug and BCVA as factors. Differences between categorical variables were tested for significance with a Chi-Square test.

Dunnett’s multiple comparison test was used to determine the significance of any change from baseline within treatment groups. A P value < 0.05 was considered statistically significant.

## Results

**Fig. 1:**
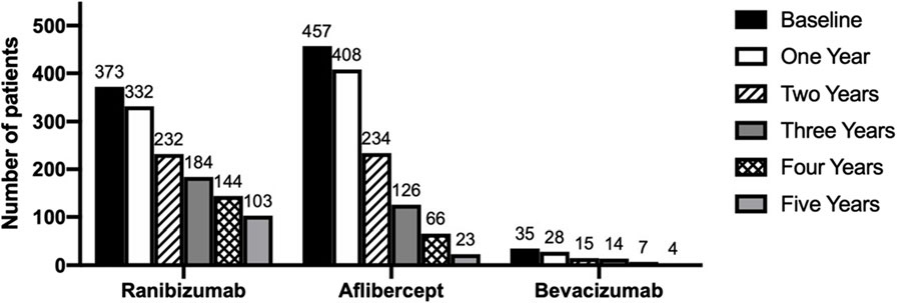
Number of patients included in the study grouped by intravitreal treatment drug. The number of patients for whom one to five years of follow-up data is available is displayed

**Fig. 2:**
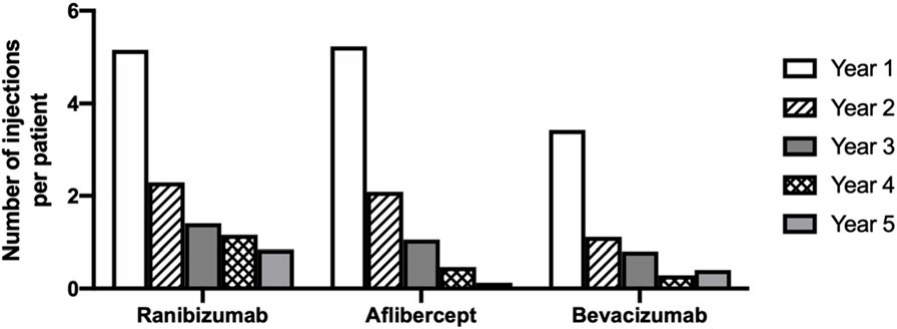
Number of injections per patient stratified by drug and year of treatment

Patient demographic data is displayed in Table 2 and Fig. 1. The mean age of the group treated with Bevacizumab was significantly lower than that of patients treated with Aflibercept (71.9 versus 82.7 years, *p* < 0.0001, Tukeys’ multiple comparison test) which was in turn significantly lower than the mean age of the Ranibizumab group (85.5 years, p = 0.0003, Tukeys’ multiple comparison test). The gender distribution was not significantly different between the groups. One-year follow-up data were available for 82.5% of patients, whilst follow-up data was available for 55.6%, 37.6%, 25.1%, and 15.0% of the cohort at years 2, 3, 4, and 5 respectively. Figure 2 shows the mean number of injections per eye per year of Ranibizumab, Aflibercept, or Bevacizumab treatment. No significant difference in the number of injections per year was detected between the groups treated with Ranibizumab and Aflibercept (*p* = 0.365, Tukey’s multiple comparison test), or between patients treated with Aflibercept and Bevacizumab (*p* = 0.123). The Bevacizumab group, however, received significantly fewer injections on average than those treated with Ranibizumab (*p* = 0.015, Tukey’s multiple comparison test).

**Table 2:** Baseline demographics and visual acuity of the eyes diagnosed with wet AMD and treated with either Ranibizumab, Aflibercept or Bevacizumab

*Baseline*	**Ranibizumab**	**Aflibercept**	**Bevacizumab**	***P***
Number of eyes (patients)	373 (336)	457 (412)	35 (32)	-
Age (years, mean)	85.5 (± 8.65)	82.7 (± 10.40)	71.9 (± 19.04)	< *0.0001*
Sex—male (%)	149 (39.9%)	191 (41.8%)	13 (37.1%)	0.74
Best Corrected Visual Acuity (BCVA, LogMAR)	0.59 (± 0.22)	0.56 (± 0.21)	0.62 (± 0.46)	0.53

**Fig. 3:**
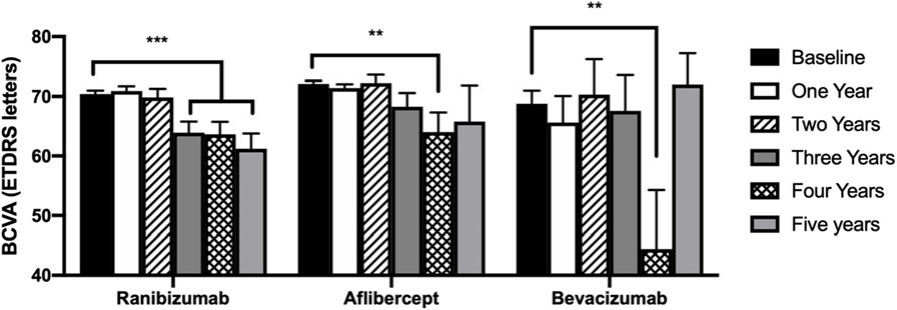
Mean ± SEM BCVA expressed as an ETDRS letter score over the five-year study period for patients treated with Ranibizumab, Aflibercept, and Bevacizumab. **p < 0.01 versus baseline, ***p < 0.001 versus baseline, Dunnett’s multiple comparison test

**Fig. 4:**
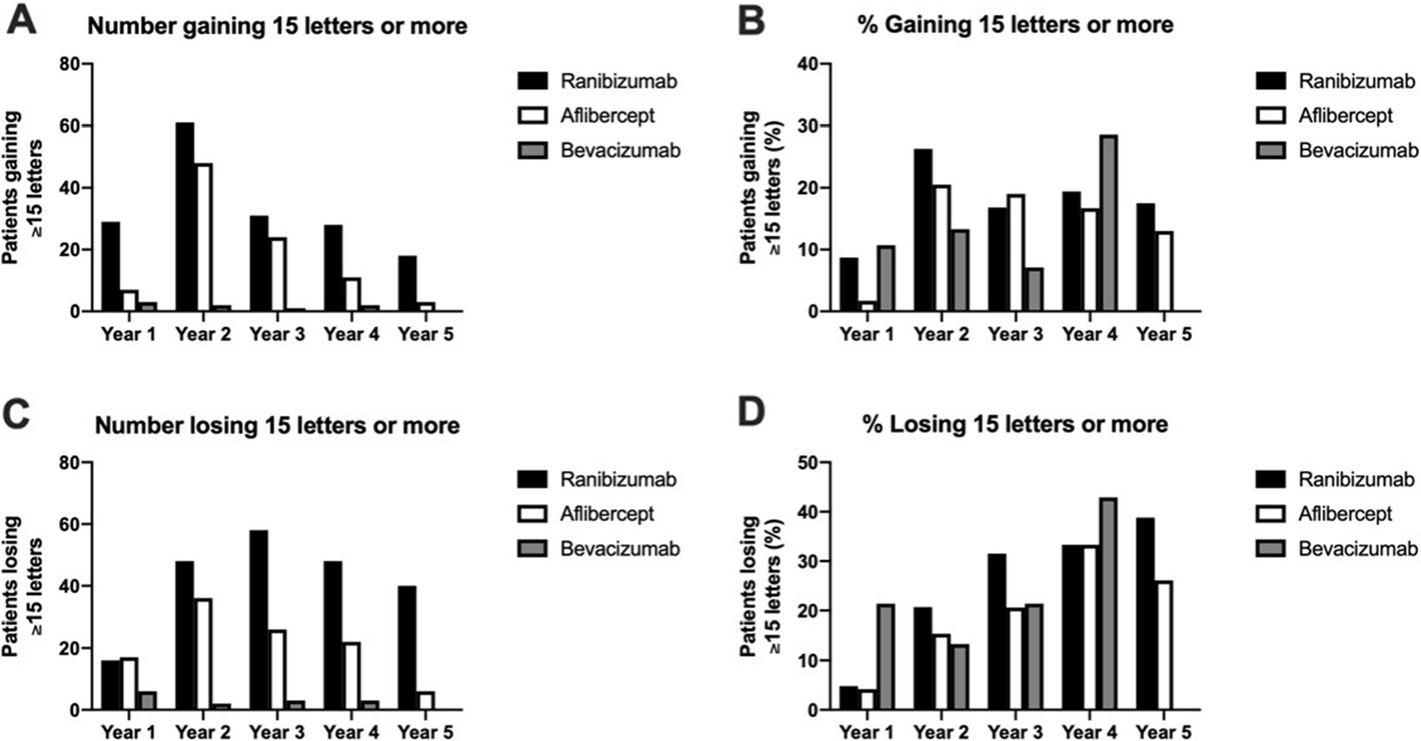
Visual gains and losses in the cohort stratified by treatment drug. (**A**) Number of patients gaining 15 or more ETDRS letters from baseline per treatment year. (**B**) Percentage of patients gaining 15 or more ETDRS letters from baseline per treatment year. (**C**) Number of patients losing 15 or more ETDRS letters from baseline per treatment year. (**B**) Percentage of patients gaining 15 or more ETDRS letters from baseline per treatment year

**Fig. 5:**
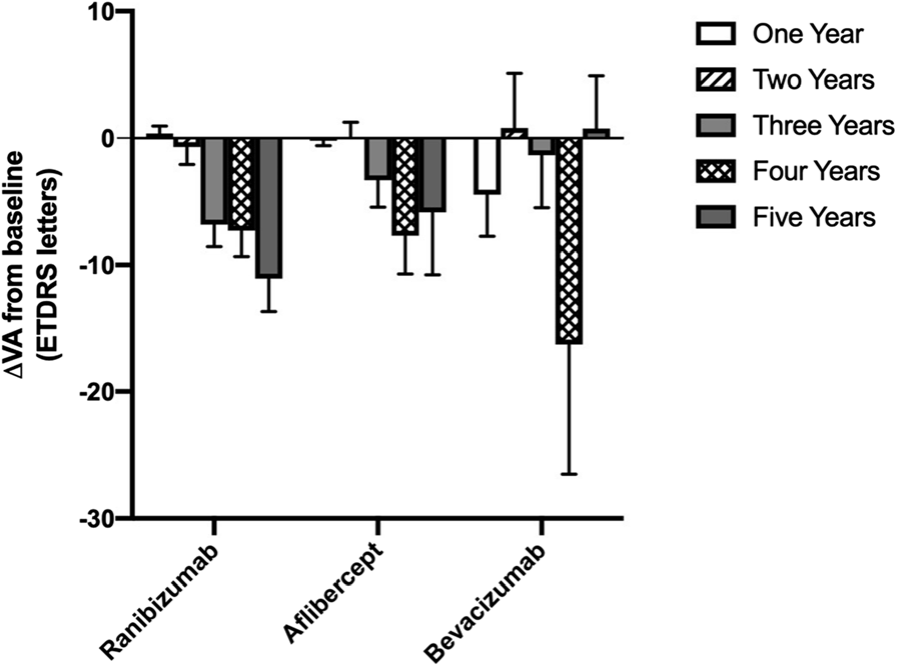
Mean ± SEM change in BCVA from baseline measured in ETDRS letters for patients treated with Ranibizumab, Aflibercept and Bevacizumab

Baseline BCVA measured in the number of letters read on the ETDRS chart is shown in Table 2. No significant difference in mean baseline BCVA was detected between the three groups (*p* > 0.05 for all comparisons, Tukey’s multiple comparison test). Mean BCVA in patients treated over the time period of the study is shown in Fig. 3 for each of the three drugs. The number and percentage of patients who either gained or lost 15 letters or more for each year of the study are displayed in Fig. 4. No significant differences in these metrics were detected between the three groups over the period considered (Two way ANOVA F(2, 8) = 0.932, *p* = 0.43 for the proportion gaining 15 or more letters; F(2, 8) = 0.465, *p* = 0.64 for the proportion losing 15 or more letters). Figure 5 shows the mean change in visual function over the study period for each of the three drugs patients were treated with. There was a gradual deterioration of the BCVA over time for the patient group as a whole (two way ANOVA for effect of time F (4, 1905) = 3.57, *p* = 0.007), but no difference was detected in this regard between the three molecules used (two-way ANOVA for effect of drug F(2, 1905) = 1.03, *p* = 0.36, interaction drug x time F(8, 1905) = 1.10, *p* = 0.36).

## Discussion

To the best of our knowledge, this is the first retrospective real-life clinical study in which three different anti-VEGF agents for wet-AMD have been compared over a long-term follow-up period.

In the literature, there are only short-term studies (1 or 2 years of follow-up) confirming substantial equality in the effectiveness of the different molecules.

Martin et al. studied the effects of Ranibizumab and Bevacizumab when administered either monthly or as needed for two years [20]. The authors concluded that mean gain in visual acuity was similar for both drugs among patients following the same regimen for two years. However, small differences in mean gain in visual acuity emerged at year 2 when comparing the 2 dosing regimens; as-needed dosing of either drug resulted in 2.4 letters less mean gain than that seen with monthly dosing.

These results are supported by a recent meta-analysis of all comparative trials, which showed essentially no difference between Bevacizumab and Ranibizumab in mean change in visual acuity after 1 year of treatment [21].

A separate clinical trial comparing the effect of Aflibercept and Ranibizumab showed an equivalent visual gain between Aflibercept injected every 8 weeks and Ranibizumab injected every 4 weeks [11, 22].

Furthermore, a Cochrane review published in 2019 examined data from 16 randomized clinical trials (RCTs) conducted worldwide with Ranibizumab, Bevacizumab, and Pegaptanib [23]. This review found that visual acuity was improved after one year of treatment and follow-up. Regarding longer term results, other RCTs have reported a VA increase at 1- and 2-years follow-up of wet AMD patients treated with intravitreal anti-VEGF agents [24–26].

However, whilst clinical outcomes from the first 1 to 2 years of anti-VEGF treatment have been well documented by large-scale clinical trials, relatively few investigators have addressed outcomes after 3 or more years [27–29]. Maguire and colleagues assigned patients randomly to 1 of 4 treatment groups defined by drug (Ranibizumab or Bevacizumab) and by dosing regimen (monthly or as-needed). They concluded that even though the vision was conserved in their cohort over the first 2 years of treatment with both molecules, these visual gains were not maintained at 5 years, with a progressive deterioration observed in further follow-up after 2 years. There were no obvious differences in visual acuity outcomes at year 5 between the two different treatment regimens [28]. Another recent review analyzed the long-term outcomes (36 months) of six prospective extension/follow-up studies and five retrospective studies [30]. Significant improvements in VA were observed in the first few years of anti-VEGF therapy, followed by a gradual decline in most studies. In the studies selected for this review of long-term outcomes, patients were mainly treated with an as-needed regime, whilst 3 of the studies provided consistent dosing in the form of a fixed or a treat-and-extend protocol. A consensus from these publications seems to be that the greater frequency of injections in consistent dosing regimens leads to superior VA outcomes compared to as-needed dosing regimens [27].

In our study, we retrospectively evaluated three different cohorts of patients, showing real-life treatment outcomes. Our patients were treated with Ranibizumab, Bevacizumab, or Aflibercept over a maximum follow-up of 5 years. One of the strengths of our study was that our results were obtained outside of clinical trial rules, providing real-world data on the effectiveness of anti-VEGF therapy. Because none of the drugs completely eliminate neovascularization, treatment continues indefinitely for most patients. Therefore, the 5-year-long follow-up of our study contributes important data for the long-term evaluation of these therapies.

It has been reported that the different treatment regimens adopted for intravitreal therapy in wet AMD can lead to high variability in BCVA results at 2 years [26]. Therefore, we used a TEX regime for all patients, in line with recommendations from the UK Retinal Outcomes Group [31], who concluded that a TEX regimen constitutes the best regimen for patients. Our data showed a relative stabilization of visual function during the first 2 years of treatment for all the patients, with no significant differences detected between the different treatment molecules. The VA then appeared to deteriorate from year 3 onwards, in particular for the Bevacizumab group in which a drop in visual acuity was noticed at year four. We can speculate this result is due to the significant drop in the number of patients.

Our visual outcomes are concordant with those from Mehta et al. study, which reviewed 26 studies providing outcome data on Ranibizumab and Aflibercept. They found that the visual gains achieved in the first year of treatment were rarely maintained, and suggested that under-treatment was likely a contributing factor [32].

We are not able to speculate on macular health as we did not correlate our results with the anatomical changes on OCT. However, some authors have reported that macular atrophy affects long-term visual outcomes of anti-VEGF therapy and this may explain the gradual decline in the BCVA after year 3 noticed in our cohort [33, 34].

Regarding adverse effects of anti-VEGF treatment, Plyukhova et al. compared the safety of Bevacizumab, Aflibercept, and Ranibizumab in their review of 13 RCTs involving 4952 patients. They concluded that the safety profile of these drugs did not differ significantly. However, macular atrophy was reported heterogeneously, and it should be considered a serious adverse event [35].

It has also been reported that visual gain correlates positively with the number of injections given [27]. Adrean et al. in 2018 analyzed data from patients that had received at least 50 injections, with a mean follow-up of 8 years, and found that over 35% of their patient cohort had an improvement of 15 letters (3 lines) or more with a TEX regime [36]. This is a substantially greater proportion than the equivalent subset of patients in our study. In addition, we found that in our cohort patients treated with Bevacizumab received a lower number of injections per year compared to the other two groups of patients, whilst the average number of yearly injections of Ranibizumab and Aflibercept did not statistically significantly differ. This is probably explained by the fact that Bevacizumab was used off-label only in patients with advanced or early stages of macular degeneration with very low or very good visual function. The number of injections reported in our study differed significantly from the average amount of injections reported in clinical trials. However, our findings are in line with a previously published real-life study by Gayadine-Harricham et al. and with a review by Metha et al. showing huge variability of this parameter in previous studies ranging from 4.5 to 11.4 injections during the first year of treatment [32, 33].

Other long-term studies have found that at five years, a third of treated eyes lost 15 ETDRS letters (3 lines) or more [28, 29]. This is broadly concordant with the results of our study (Fig. 4), as are the results of Engelbert et al., who published data suggesting visual acuity preservation and stabilization occurred for up to 36 months [37].

The current study is subject to some limitations, including variations in the mean age and size of the 3 groups of patients. This is because Bevacizumab was only used in patients with relatively preserved visual function (presenting with early stages of disease) or very low visual function (due to a more advanced stage of disease), who were more likely to be older patients. Because of the real-life design of the study, we chose to include the Bevacizumab group and their data despite the potential source of bias.

The treatment stop or the loss at follow-up caused numerous deviations from treatment regimen as previously reported by Boyle et al. [38]. In addition, there were patients with no minimal data requirements for analysis at different time-points. This has resulted in missing data and relatively few patients with long-term follow-up data, especially after 3 years. Our dataset did not allow us to provide detailed information about the percentage of patients deviated from the TEX regimen and the cause (e.g. discontinued treatment, loss of follow up, missing data). Thus, further real-life studies investigating the numerous and different causes of poor compliance with the appointments and with the treatment regimen, and investigating the rate of discontinuation of treatment are needed.

The presence of 97 patients with only 1 year of follow-up data available may introduce bias in our results, but these patients were included in our statistical analysis in order to reflect the real-life nature of the paper. Finally, we have not stratified and analyzed therapeutic responses in different types of CNV (classical and occult), although published literature has found that outcomes between subtypes may not significantly differ after 5 years of anti-VEGF therapy [39].

Further studies that correlate morphological data with functional data and try to explain the alterations underlying visual changes over time are important future avenues of research, as is the incidence of adverse events in a real-life setting.

In conclusion, we did not find any significant differences between therapy with 3 different anti-VEGF molecules in patients with wet AMD in real-life conditions. Despite long-term treatment, we found a slight reduction in visual function occurred after the third year of follow-up.

## Data Availability

The datasets used and/or analysed during the current study are available from the corresponding author on reasonable request.

## References

[1] Congdon N, O’Colmain B, Klaver CC, Klein R, Muñoz B, Friedman DS (2004). Causes and prevalence of visual impairment among adults in the United States. Arch Ophthalmol.

[2] Bourne RR, Stevens GA, White RA, Smith JL, Flaxman SR, Price H, Jonas JB, Keeffe J, Leasher J, Naidoo K, Pesudovs K, Resnikoff S, Taylor HR; Vision Loss Expert Group. Causes of vision loss worldwide, 1990-2010: a systematic analysis. Lancet Glob Health. 2013;1(6):e339–49. 10.1016/S2214-109X(13)70113-X 25104599

[3] Bressler NM (2004). Age-related macular degeneration is the leading cause of blindness. JAMA.

[4] Gass JD, Agarwal A, Lavina AM, Tawansy KA (2003). Focal inner retinal hemorrhages in patients with drusen: an early sign of occult choroidal neovascularization and chorioretinal anastomosis. Retina.

[5] Senger DR, Galli SJ, Dvorak AM, Perruzzi CA, Harvey VS, Dvorak HF (1983). Tumor cells secrete a vascular permeability factor that promotes accumulation of ascites fluid. Science.

[6] Miller JW, Adamis AP, Aiello LP. Vascular endothelial growth factor in ocular neovascularization and proliferative diabetic retinopathy. Diabetes Metab Rev. 1997;13(1):37–50. 10.1002/(sici)1099-0895(199703)13:1<37::aid-dmr174>3.0.co;2-k 9134347

[7] Ng EW, Adamis AP. Targeting angiogenesis, the underlying disorder in neovascular age-related macular degeneration. Can J Ophthalmol. 2005;40(3):352–68. 10.1016/S0008-4182(05)80078-X 15947805

[8] Rosenfeld PJ, Brown DM, Heier JS, Boyer DS, Kaiser PK, Chung CY, Kim RY; MARINA Study Group. Ranibizumab for neovascular age-related macular degeneration. N Engl J Med. 2006;355(14):1419–31. 10.1056/NEJMoa054481 17021318

[9] Tufail A, Patel PJ, Egan C, Hykin P, da Cruz L, Gregor Z, Dowler J, Majid MA, Bailey C, Mohamed Q, Johnston R, Bunce C, Xing W; ABC Trial Investigators. Bevacizumab for neovascular age related macular degeneration (ABC Trial): multicentre randomised double masked study. BMJ. 2010;340:c2459. 10.1136/bmj.c2459 20538634

[10] Tufail A, Xing W, Johnston R (2014). The neovascular age-related macular degeneration database: multicenter study of 92 976 ranibizumab injections: report 1: visual acuity. Ophthalmology.

[11] Schmidt-Erfurth U, Kaiser PK, Korobelnik JF, Brown DM, Chong V, Nguyen QD, Ho AC, Ogura Y, Simader C, Jaffe GJ, Slakter JS, Yancopoulos GD, Stahl N, Vitti R, Berliner AJ, Soo Y, Anderesi M, Sowade O, Zeitz O, Norenberg C, Sandbrink R, Heier JS (2014). Intravitreal aflibercept injection for neovascular age- related macular degeneration: ninety-six-week results of the VIEW studies. Ophthalmology.

[12] Dugel PU, Jaffe GJ, Sallstig P, Warburton J, Weichselberger A, Wieland M, Singerman L (2017). Brolucizumab Versus Aflibercept in Participants with Neovascular Age-Related Macular Degeneration: A Randomized Trial. Ophthalmology.

[13] European Medicines Agency. Workshop on Identifying Opportunities for “big Data” in Medicines Development and Regulatory Science. E.M.A, 2016. https://www.ema.europa.eu/en/documents/report/report-workshop-identifying-opportunities-big-data-medicines-development-regulatory-science_en.pdf. Accessed 2 Aug 2021.

[14] Use of Real-world Evidence to Support Regulatory Decision-making for Medical Devices: Draft Guidance for Industry and Food and Drug Administration Staff. F.D.A, 2016. https://www.fda.gov/media/99447/download. Accessed 2 Aug 2021.

[15] Ranibizumab and Pegaptanib for the Treatment of Age-related Macular Degeneration. NICE. 2008. https://www.nice.org.uk/guidance/ta155. Accessed 2 Aug 2021.

[16] Aflibercept Solution for Injection for Treating Wet Age-related Macular Degeneration. NICE. 2013. https://www.nice.org.uk/guidance/ta294. Accessed 2 Aug 2021.

[17] Liew G, Lee AY, Zarranz-Ventura J, Stratton I, Bunce C, Chakravarthy U, Lee CS, Keane PA, Sim DA, Akerele T, McKibbin M, Downey L, Natha S, Bailey C, Khan R, Antcliff R, Armstrong S, Varma A, Kumar V, Tsaloumas M, Mandal K, Egan C, Johnston RL, Tufail A (2016). The UK Neovascular AMD Database Report 3: inter-centre variation in visual acuity outcomes and establishing real-world measures of care. Eye (Lond).

[18] Butt T, Tufail A, Rubin G (2017). Health state utility values for age-related macular degeneration: review and advice. Appl Health Econ Health Pol.

[19] Corazza P, Kabbani J, Soomro T, Alam MMR, D'Alterio FM, Younis S. Three-year real-world outcomes of intravitreal anti-VEGF therapies in patients affected by myopic choroidal neovascularization. Eur J Ophthalmol. 2020:1120672120963455. 10.1177/1120672120963455 33135487

[20] Comparison of Age-related Macular Degeneration Treatments Trials (CATT) Research Group, Martin DF, Maguire MG, Fine SL, Ying GS, Jaffe GJ, Grunwald JE, Toth C, Redford M, Ferris FL 3rd. Ranibizumab and bevacizumab for treatment of neovascular age-related macular degeneration: two-year results. Ophthalmology. 2012;119(7):1388-98. 10.1016/j.ophtha.2020.01.029 32200813

[21] Heier JS, Brown DM, Chong V, Korobelnik JF (2012). Intravitreal aflibercept (VEGF trap-eye) in wet age-related macular degeneration. Ophthalmology.

[22] Arevalo JF, Lasave AF, Wu L, Acón D, Berrocal MH, Diaz-Llopis M, Gallego-Pinazo R, Serrano MA, Alezzandrini AA, Rojas S, Maia M, Lujan S; Pan-American Collaborative Retina Study Group (PACORES). INTRAVITREAL BEVACIZUMAB FOR CHOROIDAL NEOVASCULARIZATION IN AGE-RELATED MACULAR DEGENERATION: 5-Year Results of The Pan-American Collaborative Retina Study Group. Retina. 2016;36(5):859–67. 10.1097/IAE.0000000000000827 26529555

[23] Solomon SD, Lindsley K, Vedula SS, Krzystolik MG, Hawkins BS. Anti-vascular endothelial growth factor for neovascular age-related macular degeneration. Cochrane Database Syst Rev. 2014;8(8):CD005139. 10.1002/14651858.CD005139.pub3 PMC4270425 25170575

[24] Brown DM, Michels M, Kaiser PK, Heier JS, Sy JP, Ianchulev T; ANCHOR Study Group. Ranibizumab versus verteporfin photodynamic therapy for neovascular age-related macular degeneration: Two-year results of the ANCHOR study. Ophthalmology. 2009;116(1):57–65.e5. 10.1016/j.ophtha.2008.10.018 19118696

[25] Abraham P, Yue H, Wilson L. Randomized, double-masked, sham-controlled trial of ranibizumab for neovascular age-related macular degeneration: PIER study year 2. Am J Ophthalmol. 2010;150(3):315–324.e1. 10.1016/j.ajo.2010.04.011 20598667

[26] Ho AC, Busbee BG, Regillo CD, Wieland MR, Van Everen SA, Li Z, Rubio RG, Lai P; HARBOR Study Group. Twenty-four-month efficacy and safety of 0.5 mg or 2.0 mg ranibizumab in patients with subfoveal neovascular age-related macular degeneration. Ophthalmology. 2014;121(11):2181–92. 10.1016/j.ophtha.2014.05.009 25015215

[27] Qin VL, Young J, Silva FQ (2018). Outcomes of patients with exudative age-related macular degeneration treat with antivascular endothelial growth factor therapy for three or more years: A review of current outcomes. Retina.

[28] Comparison of Age-related Macular Degeneration Treatments Trials (CATT) Research Group, Maguire MG, Martin DF, Ying GS, Jaffe GJ, Daniel E, Grunwald JE, Toth CA, Ferris FL 3rd, Fine SL. Five-Year Outcomes with Anti-Vascular Endothelial Growth Factor Treatment of Neovascular Age-Related Macular Degeneration: The Comparison of Age-Related Macular Degeneration Treatments Trials. Ophthalmology. 2016;123(8):1751–61. 10.1016/j.ophtha.2016.03.045 PMC4958614 27156698

[29] Rofagha S, Bhisitkul RB, Boyer DS, Sadda SR, Zhang K; SEVEN-UP Study Group. Seven-year outcomes in ranibizumab-treated patients in ANCHOR, MARINA, and HORIZON: a multicenter cohort study (SEVEN-UP). Ophthalmology. 2013;120(11):2292–9. 10.1016/j.ophtha.2013.03.046 23642856

[30] Singer MA, Awh CC, Sadda S, Freeman WR, Antoszyk AN, Wong P, Tuomi L. HORIZON: an open-label extension trial of ranibizumab for choroidal neovascularization secondary to age-related macular degeneration. Ophthalmology. 2012;119(6):1175–83. 10.1016/j.ophtha.2011.12.016 22306121

[31] Amoaku W, Balaskas K, Cudrnak T, Downey L, Groppe M, Mahmood S, Mehta H, Mohamed Q, Mushtaq B, Severn P, Vardarinos A, Yang Y, Younis S. Initiation and maintenance of a Treat-and-Extend regimen for ranibizumab therapy in wet age-related macular degeneration: recommendations from the UK Retinal Outcomes Group. Clin Ophthalmol. 2018;12:1731–40. 10.2147/OPTH.S174560 PMC6136415 30237693

[32] Mehta H, Kim LN, Mathis T, Zalmay P, Ghanchi F, Amoaku WM, Kodjikian L (2020). Trends in Real-World Neovascular AMD Treatment Outcomes in the UK. Clin Ophthalmol.

[33] Gayadine-Harricham Y, Rufin V, Law-Koune S, Tran THC (2020). Four-Year Outcome of Aflibercept Treatment-Naïve Patients for Neovascular Age-Related Macular Degeneration: Evidence from a Clinical Setting. J Ophthalmol.

[34] Munk MR, Ceklic L, Ebneter A, Huf W, Wolf S, Zinkernagel MS (2016). Macular atrophy in patients with long-term anti-VEGF treatment for neovascular age-related macular degeneration. Acta Ophthalmol.

[35] Plyukhova AA, Budzinskaya MV, Starostin KM, Rejdak R, Bucolo C, Reibaldi M, Toro MD (2020). Comparative Safety of Bevacizumab, Ranibizumab, and Aflibercept for Treatment of Neovascular Age-Related Macular Degeneration (AMD): A Systematic Review and Network Meta-Analysis of Direct Comparative Studies. J Clin Med.

[36] Adrean SD, Chaili S, Ramkumar H, Pirouz A, Grant S. Consistent Long-Term Therapy of Neovascular Age-Related Macular Degeneration Managed by 50 or More Anti-VEGF Injections Using a Treat-Extend-Stop Protocol. Ophthalmology. 2018;125(7):1047–53. 10.1016/j.ophtha.2018.01.012 29439828

[37] Engelbert M, Zweifel SA, Freund KB. "Treat and extend" dosing of intravitreal antivascular endothelial growth factor therapy for type 3 neovascularization/retinal angiomatous proliferation. Retina. 2009;29(10):1424–31. 10.1097/IAE.0b013e3181bfbd46 19898180

[38] Boyle J, Vukicevic M, Koklanis K, Itsiopoulos C, Rees G (2018). Experiences of patients undergoing repeated intravitreal anti-vascular endothelial growth factor injections for neovascular age-related macular degeneration. Psychol Health Med.

[39] Invernizzi A, Nguyen V, Teo K, Barthelmes D, Fung A, Vincent A, Gillies M. Five-Year Real-World Outcomes of Occult and Classic Choroidal Neovascularization: Data From the Fight Retinal Blindness! Project. Am J Ophthalmol. 2019;204:105–12. 10.1016/j.ajo.2019.03.001 30862501

